# CDK9 inhibitors in acute myeloid leukemia

**DOI:** 10.1186/s13046-018-0704-8

**Published:** 2018-02-23

**Authors:** Silvia Boffo, Angela Damato, Luigi Alfano, Antonio Giordano

**Affiliations:** 1 Sbarro Institute for Cancer Research and Molecular Medicine, Department of Biology, Temple University, 1900 N. 12th St., Room 431, Philadelphia, PA 19122-6017 USA; 2 Medical Oncology Unit, Clinical Cancer Centre, IRCCS–Arcispedale S. Maria Nuova, Reggio Emilia, Italy; 3 Oncology Research Center of Mercogliano (CROM), Istituto Nazionale Per Lo Studio E La Cura Dei Tumori “Fondazione Giovanni Pascale”, IRCCS, Naples, Italy; 4 Department of Medicine, Surgery, and Neuroscience, University of Siena, Siena, Italy

**Keywords:** Acute myeloid leukemia, CDK9 inhibitor, Positive transcription elongation factor b, P-TEFb, *MCL-1*, *MYC*

## Abstract

Current treatment for acute myeloid leukemia (AML) is less than optimal, but increased understanding of disease pathobiology and genomics has led to clinical investigation of novel targeted therapies and rational combinations. Targeting the cyclin-dependent kinase 9 (CDK9) pathway, which is dysregulated in AML, is an attractive approach. Inhibition of CDK9 leads to downregulation of cell survival genes regulated by super enhancers such as *MCL-1*, *MYC*, and cyclin D1. As CDK9 inhibitors are nonselective, predictive biomarkers that may help identify patients most likely to respond to CDK9 inhibitors are now being utilized, with the goal of improving efficacy and safety.

**Electronic supplementary material:**

The online version of this article (10.1186/s13046-018-0704-8) contains supplementary material, which is available to authorized users.

## Background

Acute myeloid leukemia (AML) is a heterogeneous hematologic malignancy characterized by a clonal proliferation of immature myeloid precursor cells in the peripheral blood, bone marrow, and/or other tissues. It is the most common acute adult leukemia, with approximately 21,380 individuals in the United States diagnosed in 2017 [1]. AML is primarily a disease of older adults, with a median age at diagnosis of 68 years. It may develop de novo or secondarily due to progression of myelodysplastic syndrome (MDS) or chronic bone marrow stem cell disorders [2] or as a result of prior cytotoxic chemotherapy, particularly alkylating agents and topoisomerase inhibitors [3].

Treatment for AML has been less than optimal. The standard induction regimen, a continuous infusion of cytarabine for 7 days plus 3 days of an anthracycline, usually daunorubicin or idarubicin (7 + 3 therapy), has changed little over the past 40 years. Complete remission (CR) rates rarely top 70% in younger patients and 50% in older patients [4], and overall 5-year survival is only 27% [1]. Over the 3 decades from 1977 to 2006, there has been a modest improvement in overall survival for patients aged 64 to 75 years, but not for those 75 years or older [5]. The prognosis for primary refractory and relapsed or refractory (R/R) AML is particularly poor [6, 7]. After first relapse, 1- and 5-year survival rates of 29% and 11%, respectively, have been reported [7]. These poor outcomes necessitate new treatment options for the disease, including those that overcome drug resistance.

An increasing understanding of the pathobiology and genomics of AML has led to clinical investigation of a variety of novel therapeutic approaches, particularly agents targeted against dysregulated enzymes and mutant driver proteins. In addition, investigations into mechanisms of drug resistance in AML have shed light on means of overcoming chemoresistance, such as targeting leukemic stem cells and the bone marrow microenvironment [8–10]. Two new targeted agents were approved by the US Food and Drug Administration (FDA) in 2017, representing the first new AML drugs available since 2000. Midostaurin, a small molecule kinase inhibitor, was approved for use in combination with standard cytarabine and daunorubicin induction and cytarabine consolidation chemotherapy for the treatment of adult patients with newly diagnosed *FLT3*-mutated AML [11]. Enasidenib, an oral targeted inhibitor of the isocitrate dehydrogenase-2 (IDH2) enzyme, was approved for the treatment of adult patients with R/R AML with an *IDH-2* mutation as detected by an FDA-approved test [12]. In addition, gemtuzumab ozogamicin, which originally received accelerated approval in 2000 but was voluntarily withdrawn from the market, was also approved for the treatment of adults with newly diagnosed CD33-positive AML and for patient 2 years and older with R/R CD33-positive AML [13]. In addition to these targeted agents, a liposome-encapsulated combination of daunorubicin and cytarabine was approved for the treatment of adults with newly diagnosed therapy-related AML or AML with myelodysplasia-related changes, both of which have a poor prognosis [14].

A therapeutic target that has been investigated in AML is cyclin-dependent kinase (CDK)9, one of a large number of CDKs that control cell-cycle progression and gene transcription. Although originally thought to act via cell-cycle regulation, CDK9 is involved in regulating gene transcription elongation and messenger RNA (mRNA) maturation, as well as other physiologic processes [15, 16]. Dysregulation in the CDK9 pathway has been observed in AML and other hematologic malignancies and in solid tumors, making it an attractive target for cancer therapeutics [17]. In this review, we provide an updated overview of the biology of CDK9 and describe the role of the CDK9 pathway in AML, providing rationale supporting its use as a therapeutic target. This is followed by a review of CDK9 inhibitors in clinical and preclinical development for AML and other hematologic malignancies.

### Biology of CDK9

Together with regulatory subunits (cyclins), CDKs form functional complexes responsible for the control of cell proliferation, differentiation, apoptosis, and DNA repair [17]. Whereas many CDKs (eg, CDK1, CDK2, CDK3, CDK4, and CDK6) control cell-cycle progression, ensuring timely and accurate cell replication, others (ie, CDK8 and CDK9) function as gene transcription controllers [18]. CDK9 plays a critical role in controlling global (non-ribosomal) transcription, notably including expression of genes that are regulated by super enhancers, large clusters of DNA regulatory elements (“enhancers”) that drive transcription of genes involved in cell identity [19]. Such genes include *MYC*, a downstream proto-oncogene involved in cell growth and cell-cycle progression, and *MCL-1,* an apoptosis regulator. CDK9 also appears to be involved in several physiologic processes in the cell outside of transcription, including differentiation, apoptosis, and signal transduction [15].

CDK9 was first designated PITALRE based on a characteristic amino acid motif (Pro-Ile-Thr-Ala-Leu-Arg-Glu), and its function was first elucidated in studies of human immunodeficiency virus [20, 21]. CDK9 exists in two isoforms, the originally identified major 42 kDa protein (CDK9_42_) and a minor 55 kDa (CDK9_55_) protein that is translated from an in-frame mRNA that arises from an upstream transcriptional start site [22, 23].

**Fig. 1:**
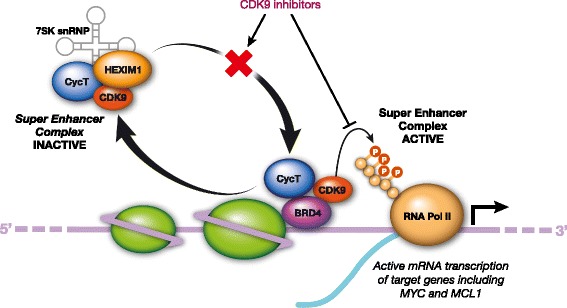
Role of cyclin-dependent kinase (CDK)9 in gene transcription and cancer cell survival. CDK9 associates with cyclin T1 (CycT), forming the positive transcription elongation factor b (P-TEFb) complex that regulates gene transcription elongation and mRNA maturation [15]. The P-TEFb complex remains inactive when bound to hexamethylene bisacetamide-inducible protein 1 (HEXIM1), which is associated with the noncoding 7SK small nuclear RNA (snRNA) [45]. Bromodomain protein 4 (BRD4) recruits P-TEFb to activate the complex and transcription. CDK9 phosphorylates the carboxyl terminal domain of RNA polymerase II (RNA Pol II), allowing transcriptional elongation and expression of genes such as *MYC* and *MCL-1,* which together increase proliferation and survival of cancer cells

Both CDK9 isoforms generate a heterodimer with regulatory cyclins T1, T2a, or T2b to form the main component of the positive transcription elongation factor b (P-TEFb) complex that stimulates transcription elongation by phosphorylating the carboxy-terminal domain (CTD) of the largest subunit of RNA polymerase II (RNA Pol II); the CTD contains tandem repeats of a 7 amino-acid sequence that is phosphorylated by CDK7 at Ser5 (YSPT**Ser5**PS) and CDK9 at Ser2 (Y**Ser2**PTSPS). Ser5 phosphorylation results in activation of RNA Pol II such that transcription is initiated and Ser2 phosphorylation allows productive transcriptional elongation (Fig. 1 [15]). Therefore CDK9 inhibition prevents productive transcription and is associated with a global reduction in mRNA, including genes, such as *MYC* and *MCL-1,* which regulate proliferation and survival of cancer cells [15, 24–26]. Cyclin K can also interact with CDK9 isoforms in vitro and in vivo, and the CDK9–cyclin K complex can activate transcription when tethered to RNA, but not to DNA, in vitro [24]. Further investigations have shown that CDK9 is involved in co-transcriptional histone modification, mRNA processing, mRNA export, and DNA repair [16, 27, 28].

Although their phosphorylation patterns may be identical, the CDK9 isoforms display differences in subcellular localization and expression patterns, regulation, and tissue distribution [23, 28]. CDK_42_ has been reported to localize to the nucleoplasm whereas CDK9_55_ localizes to the nucleolus [23, 26]. Also, CDK_55_, but not CDK9_42_, specifically associates with Ku70 and appears to play a role in DNA repair and cell viability through a distinct function [28]. In addition, there are numerous reports of differential expression patterns, including in hematologic cells [25]. Interestingly, CDK9_55_ is preferentially expressed following induced differentiation of human primary monocytes into macrophages [23], whereas stimulation of human macrophages with lipopolysaccharide, or infection with the human immunodeficiency virus type 1 (HIV-1) increases CDK9_42_ expression [29]. Also activation of primary lymphocytes results in increased CDK9_42_ and decreased CDK9_55_ expression [23]. Taken together, these findings suggest that the function of the two CDK9 isoforms is likely to be at least partially distinct, although further studies are required to produce definitive evidence [26] and importantly in the cancer context, including in AML.

### Role of the CDK9 pathway in cancers, including AML

The CDK9-related pathway has emerged as a prioritized target for cancer therapy across a range of tumor types [30]. Multiple studies have shown that a dysregulated CDK9 signaling system may have important implications in the development and/or maintenance of a malignant cell phenotype [30]. Dysregulation of the CDK9 pathway has been observed in a variety of human tumors, which may induce increased expression and/or hyperactivity of cellular oncogenic factors. Studies on cancers, such as lymphoma [31, 32], prostate cancer [33], neuroblastoma [34], and other malignancies [35], demonstrate that CDK9-related pathways are dysregulated, suggesting that CDK9 overexpression promotes the cell proliferation and the synthesis of antiapoptotic factors like MCL-1, BCL,-2 and XIAP [36], which are determinants for cancer-cell survival [37]. The levels of gene products with short half-lives, such as *MYC* and *MCL-1*, are reduced most rapidly on exposure to CDK9 inhibitors, thereby leading to reduced cell proliferation and survival.

Regarding CDK9 expression in AML, the HemaExplorer [38] curated database of processed mRNA gene expression profiles provides accessible data on CDK9 expression in hematopoietic cells at different maturation stages. Expression data from distinct subtypes of human AML, defined by karyotypes, are included in the database allowing researchers to directly compare gene expression of leukemic cells with those of their closest normal counterparts. Such data point to a numeric increase in CDK9 mRNA expression in AML samples relative to common myeloid progenitor cells and also illustrates some variation in expression between AML subtypes.

However, it is important to note that overall CDK9 pathway activity is a critical aspect of dysregulation rather than CDK9 expression only. Critically, CDK9-mediated transcription of MCL-1 and MYC plays an important role in growth and survival of cancer cells, and dysregulation of this component of the CDK9 pathway is prominent in a number of hematologic malignancies [17]. The MCL-1 and MYC aspects of the CDK9 pathway have been associated with the pathogenesis of AML. For example, various translocation products of the *MLL* gene found in leukemias such as AML associate with P-TEFb and constitutively activate transcription [17, 39]. High *MCL-1* expression is linked to AML development in murine models [40], and *MCL-1* plays a key role in survival and expansion of murine and human AML cells [41]. *MCL-1* is also upregulated in about half of cases of R/R AML and is associated with a poor prognosis [42]. Levels of the P-TEFb inhibitor hexamethylene bisacetamide-inducible protein 1 (HEXIM1) are upregulated during differentiation of murine leukemia cells [43], and HEXIM1 has been shown to be involved in the tumorigenesis of AML cell lines bearing the *NPMc +* mutation, the cytoplasmic-mislocated mutant form of *NPM* seen in approximately 35% of patients with AML [44]. In addition, HEXIM1 mRNA overexpression is almost mutually exclusive with *MYC* overexpression in primary AML samples, suggesting that HEXIM1 plays a key role in the growth inhibition and apoptosis of AML cells [45].

Such disease linkage evidence encouraged studies evaluating the potential of CDK9 inhibitors as anticancer therapeutics, initially in pre-clinical models. CDK9 inhibition has been reported to lead to apoptosis in a variety of leukemia and solid tumor cell lines. Notably, the most sensitive cancer cells lines included hematologic tumor cells, especially AML [46], thereby providing functional evidence for the dependency of AML on the CDK9 pathway.

Given CDK9 is a kinase it is considered as a relatively tractable target for drug discovery and provides a route for the indirect targeting of MCL-1 and MYC that may be considered as currently more challenging targets in drug discovery [32].

### CDK9 inhibitors in clinical trials in AML and other hematologic cancers

CDK9 inhibitors have been investigated as therapeutics for a variety of hematologic cancers and solid tumors. Table 1 [46–59] provides CDK inhibition profiles for CDK9 inhibitors that have reached the clinic or have been evaluated in preclinical studies in AML and other hematologic cancers (Additional file 1: Table S1 summarizes the clinical status of other CDK9 inhibitors across a broader range of tumor types). Current CDK9 inhibitors are competitive inhibitors of the ATP-binding site, which is highly conserved across the CDK family; consequently CDK9 inhibitors lack specificity and generally also inhibit other CDKs to varying extents [60, 61]. Although some progress has been made against other CDKs in discovery of allosteric inhibitors with greater selectivity potential by targeting residues outside the kinase domain (CDK12/13 [62, 63]), no such inhibitors have been described for CDK9. Although they display activity against a variety of CDKs and enzymes, CDK9 inhibitors are referred to as such because they typically exhibit increased half maximal inhibitory concentration (IC_50_) values for CDK9 compared with other CDKs/enzymes. As described in the sections that follow, CDK9 inhibitors in general exhibit a variety of effects in AML cells and in vivo models, including reduced phosphorylation of RNA Pol II; reduced levels of proteins such as MYC, MCL-1, XIAP, and cyclin D1; induction of apoptosis; and inhibition of tumor growth and prolonged survival in animal models. There is increasing interest in identifying predictive biomarkers of response to conventional and investigational-targeted therapies in AML, including CDK9 inhibitors. For example, measuring the function of B-cell lymphoma 2 (BCL-2) family proteins using BCL-2 homology domain 3 (BH3) profiling has been shown to provide useful information in discriminating AML treatment response with traditional cytarabine-based therapy and investigational AML regimens [64–68]. The underlying principle of BH3 profiling is that mitochondrial depolarization following exposure to BH3 domain peptides serves as a functional biomarker to predict cell sensitivity to individual antiapoptotic proteins [69]. For example, sensitivity of cells to the NOXA-BH3 peptide provides a direct functional measurement of MCL-1 dependency, whereas sensitivity of cells to BAD-BH3 provides a measurement of BCL-2 dependency.

**Table 1:** CDK9 inhibitors

Agent	Mode of Administration	CDK Inhibition Profile (IC_50_)	Development Stage/Indication
Alvocidib (flavopiridol) [47, 48]	Intravenous	CDK9: 6 nM CDK4: 10 nM CDK7: 23 nM CDK11: 57 nM CDK5: 110 nM	Phase 2: AML, ALL, CLL, DLBCL, MCL, MM, various lymphomas Phase 1: AML, ALL, B-cell CLL, CML, MCL, SLL, various lymphomas
AT7519 [49]	Intravenous	CDK9: *<* 10 nM CDK5: 13 nM CDK2: 47 nM CDK4: 100 nM CDK6: 179 nM	Phase 2: CLL, MCL
BAY 1143572 [50]	Oral	Not published	Phase 1: AML, ALL, DLBCL
CDKI-73 (LS-007) [51, 52]	Intravenous, oral	CDK2: 3 nM CDK9: 6 nM CDK1: 8 nM CDK4: 8 nM CDK6: 37 nM CDK7: 134 nM	Preclinical
Dinaciclib [53, 54]	Intravenous	CDK2: 1 nM CDK5: 1 nM CDK1: 3 nM CDK9: 4 nM	Phase 3: CLL^a^ Phase 2: AML^a^, ALL^a^, B-cell CLL^a^, MCL^a^, MM Phase 1: CLL, DLBCL, MM
LY2857785 [46]	Intravenous	CDK9: 11 nM CDK8: 16 nM CDK7: 246 nM	Preclinical
P276–00^b^ [55, 56]	Intravenous	CDK9: 20 nM CDK4: 63 nM CDK1: 79 nM CDK2: 224 nM	Phase 2: MCL^a^, MM Phase 1: MM
SNS-032 (BMS-387032) [57, 58]	Intravenous	CDK9: 4 nM CDK2: 38 nM CDK7: 62 nM	Phase 1: CLL, MM
TG02 [59]	Oral	CDK9: 3 nM CDK5: 4 nM CDK2: 5 nM CDK3: 8 nM CDK1: 9 nM CDK7: 37 nM	Phase 1: AML, CML, SLL

ALL, acute lymphoblastic leukemia; AML, acute myeloid leukemia; CDK, cyclin-dependent kinase; CLL, chronic lymphocytic leukemia; CML, chronic myeloid leukemia; DLBCL, diffuse large B-cell lymphoma; IC_50_, half maximal inhibitory concentration; MCL, mantle cell lymphoma; MM, multiple myeloma; SLL, small lymphocytic lymphoma

^a^Study terminated

^b^Development discontinued

#### Alvocidib (flavopiridol)

Alvocidib was the first CDK inhibitor to enter clinical trials and has been the most studied to date. Alvocidib displays potent activity against CDK9 (6 nM), in addition to activity against CDK4, CDK5, CDK7, and CDK11 [47, 48]. Although historically the mechanism of action of alvocidib was attributed to inhibition of the cell cycle at the G1 phase via targeting of CDK4/6 [70], it is now understood that its primary mechanism of action is via transcriptional regulation via CDK9/P-TEFb [71].

In vitro studies in diverse hematologic malignancies and studies on human on AML marrow blasts have shown that alvocidib reduces levels of MCL-1, BCL-2, and cyclin D1 and inhibits phosphorylation of RNA Pol II (reviewed in Karp, 2005) [72]. Based on its noted effects on the cell cycle, transcription, and apoptosis, it was surmised that alvocidib could potentiate the cytotoxicity of cycle-dependent antileukemic agents. To evaluate the potential use of alvocidib in timed sequential therapy (TST) in the clinical setting, an in vitro model was developed using primary human bone marrow cells from adults with R/R AML, acute lymphoblastic leukemia (ALL), or newly diagnosed AML with poor risk features [73]. In this model, alvocidib induced a 4.3-fold increase in apoptosis and increased the proapoptotic and cytotoxic effects of cytarabine. Subsequent studies in AML cell lines correlated rapid downregulation of *MCL-1* and a 2-fold reduction in MCL-1 levels with enhanced apoptosis [74]. Gene expression studies in leukemic blasts from adult patients with refractory AML treated with alvocidib in a phase 1 study demonstrated induced expression of *BCL-2*, which contrasts with earlier studies demonstrating downregulation of *BCL-2* expression and may represent a protective antiapoptotic response during cell-cycle arrest [75]. Alvocidib administration also resulted in downregulation of genes encoding RNA Pol II and the oncogenic transcription factors high mobility group AT-hook 1, signal transducer and activator of transcription 3, and E2F transcription factor 1, which are known to be involved in AML and other hematologic malignancies.

Alvocidib was evaluated in combination with cytarabine and mitoxantrone (FLAM) in a TST manner in multiple clinical studies in R/R AML [48, 72, 76, 77] and newly diagnosed, nonfavorable AML [76, 78–80]. A review of the safety and efficacy results from these individual studies has recently been published [80] and is beyond the scope of this review. In phase 2 trials in newly diagnosed poor-risk AML, overall CR rates of 67% to 75% were achieved, which were higher than that seen with standard 7 + 3 therapy [76, 78–80]. In general, toxicity seen with FLAM was not increased over that seen with 7 + 3 therapy, with febrile neutropenia, infection, and hepatic dysfunction being the most common Grade 3 toxicities reported in the latest study [80]. Treatment-related mortality was similar in both treatment arms in this study, but the majority of early deaths on FLAM occurred in patients *≥*60 years. Tumor lysis syndrome (TLS) has been seen following initial dosing of alvocidib in AML studies (28% incidence overall, with 2% Grade 4), necessitating appropriate prophylaxis and monitoring [80].

There are ongoing efforts to determine predictive biomarkers to allow identification of specific subsets of patients who are likely to respond to alvocidib, such as use of BH3 profiling [66]. As NOXA interacts most directly with MCL-1, these findings suggest that the AML samples that are most responsive to FLAM treatment are highly dependent on MCL-1 for survival. MCL-1 dependency was also supported by data obtained using three additional BH3 members, and these BH3 priming profiles were additive to known risk factors associated with clinical response to chemotherapy, including cytogenetic risk factors. Receiver operating characteristic curve analysis of NOXA priming, cytogenetics, and MDS history showed that the combination of these variables was highly predictive of response to FLAM (area under the concentration-time curve 0.92, *p* = 0.0002). An ongoing international biomarker-driven phase 2 study (NCT02520011) is incorporating this predictive information in identifying a subgroup of patients most likely to respond to alvocidib. The study is comparing FLAM vs. cytarabine and mitoxantrone (AM) in patients with MCL-1-dependent R/R AML as demonstrated by NOXA-BH3 priming of ≥40% by mitochondrial profiling of the bone marrow. It includes an exploratory arm evaluating patients with newly diagnosed MCL-1-dependent high-risk AML.

A phase 1, open-label, dose-escalation, safety and biomarker prediction study was recently registered. This study will explore alvocidib and standard 7 + 3 chemotherapy in patients with newly diagnosed AML (NCT03298984). Correlation between the benefit from alvocidib in combination with 7 + 3 therapy and BH3 profiling for MCL-1 dependency will be assessed as a secondary outcome.

#### Bay 1143572

BAY 1143572 displays potent CDK9/P-TEFb inhibitory activity in the nanomolar range, with inhibitory activity against other CDKs that is at least 50-fold lower [50, 81]. In in vitro models of adult T-cell leukemia/lymphoma (ATL), BAY 1143572 inhibited phosphorylation of RNA Pol II and reduced MYC and MCL-1 levels in ATL-derived and human T-lymphotropic virus 1 (HTLV-1)-transformed lines and primary ATL cells, with subsequent growth inhibition and apoptosis [50]. It also displayed antitumor activity and prolonged survival in a human ATL cell-bearing mouse model. In AML, BAY 1143572 inhibited the proliferation of 7 cell lines (both *MLL*-rearrangement positive and negative) with a median IC_50_ of 385 nM and induced apoptosis [82]. In addition, it displayed potent in vitro activity in 8 of 10 non-*MLL*-rearranged patient AML samples, including those with mutant *NPM1* or internal tandem duplication of the juxtamembrane domain-coding sequence of the *FLT3* gene (FLT3-ITD).

A phase 1 dose escalation study of BAY 1143572 in combination with granulocyte colony-stimulating factor in patients with advanced malignancies (ie, gastric cancer, triple negative breast cancer, or diffuse large B-cell lymphoma [DLBCL]; NCT01938638) has been completed, but results are yet to be reported. A phase I dose-escalation study designed to determine the safety, pharmacokinetics, and recommended phase 2 dosing of BAY 1143572 in advanced acute leukemia has completed enrollment (NCT02345382).

#### Dinaciclib (SCH 727965)

Dinaciclib is a novel and potent inhibitor of CDK1, CDK2, CDK5, and CDK9 with IC_50_ values in the low nanomolar range [53]. In in vitro studies, dinaciclib blocked thymidine DNA incorporation (IC_50_ = 4 nM) and completely suppressed retinoblastoma (Rb) phosphorylation, which correlated with induction of apoptosis. Dinaciclib exposure resulted in cell-cycle arrest in more than 100 tumor cell lines of diverse origin and across a broad range of transformed cellular backgrounds as evidenced by based on total inhibition of bromodeoxyuridine incorporation. Broad antiproliferative activity was seen across this panel of tumor cell lines, with median IC_50_ values of 11 nM. Dinaciclib has also been shown to downregulate expression of *MCL-1* and induce apoptosis in primary patient chronic lymphocytic leukemia (CLL) cells, with activity that was independent of high-risk genomic features [83].

Apoptotic and antitumor effects of dinaciclib were demonstrated in *MLL*-rearranged AML mouse models [84]. Decreased expression of *Mcl-1* was seen and overexpression of *Mcl-1* protected AML cells from dinaciclib-induced apoptosis. In mice bearing *MLL-AF9*-driven murine and human leukemias, dinaciclib exhibited potent antitumor activity and significantly prolonged survival.

Dinaciclib has been evaluated in clinical trials in various hematologic indications, with varied effectiveness. In a phase 2 study of dinaciclib monotherapy in patients with relapsed multiple myeloma (MM), 11% of patients achieved a partial response or better [54]. The most common adverse events included diarrhea, fatigue, thrombocytopenia, nausea, leukopenia, and neutropenia. Results were reported for three additional hematologic studies that were terminated early for reasons unrelated to safety or efficacy [85–87].

A phase 2 randomized study comparing dinaciclib and gemtuzumab ozogamicin in R/R AML and evaluating dinaciclib in ALL (NCT00798213) demonstrated short-lived cytoreductive activity with dinaciclib but a lack of objective clinical response in the 20 patients treated [85]. In addition, 75% of patients receiving dinaciclib experienced grade *≥* 3 treatment-related adverse events, most commonly hematologic toxicities and fatigue. Interestingly, an additional clinical toxicity reported was TLS, where metabolic changes indicative of large-scale tumor cell lysis occur [85]. While this phenomenon requires careful monitoring and management, this provides further evidence of potent anti-tumor activity underlying the cytoreductive observations, albeit currently short term in duration. This may be interpreted as illustrating the potential of CDK9-targeted therapy. Indeed, given the rapid clinical elimination of dinaciclib together with its potent cytotoxic effects observed on longer exposure in in vitro studies, evaluation of alternative clinical dosing regimens such as prolonged infusion are proposed for future studies in acute leukemia [85]. Clearly, other considerations, such as tolerability and the intended selective targeting of short-lived antiapoptotic proteins such as MCL-1 and MYC, will also need to be considered when optimizing the drug exposure period and schedule.

A randomized phase 3 study in which 42 patients with R/R CLL received treatment suggested promising antileukemic activity with dinaciclib relative to ofatumumab, an anti-CD20 monoclonal antibody (median progression-free survival of 13.7 months vs. 5.9 months, and overall response rate of 40% vs. 8.3%, respectively) [86]. The most common grade ≥ 3 adverse events experienced by patients receiving dinaciclib were neutropenia/reduced neutrophil counts/febrile neutropenia and thrombocytopenia. Limited data from five patients treated in a phase 1 study evaluating the combination of dinaciclib and rituxumab in R/R CLL showed an adverse event profile similar to that seen with dinaciclib monotherapy [87]. No results have been reported for a discontinued phase 2 study in R/R mantle cell lymphoma and B-cell CLL (NCT00871546). Dinaciclib is being evaluated in combination with pembrolizumab in R/R hematologic malignancies (ie, CLL, MM, and DLBCL) in an ongoing phase 1 trial (NCT02684617).

#### SNS-032 (BMS-387032)

SNS-032, a potent CDK9 inhibitor (4 nM) with activity against CDK2 and CDK7, was evaluated in a phase 1 and pharmacologic study in patients with advanced CLL or MM [57, 58]. Mechanism-based target modulation (ie, inhibition of CDK7 and CDK9, reduced *MCL-1* and *XIAP* expression, and apoptosis) was demonstrated, but limited clinical activity was seen and three-quarters of the patients experienced grade 3 or 4 toxicities, mainly myelosuppression [58]. In vitro studies showed that SNS-032 inhibited proliferation of AML cell lines and primary AML blasts by inducing a reduced phosphorylation of Ser2, leading to RNA Pol II pausing and resulting in Ser5 dephosphorylation after a period of time [88]. Combining SNS-032 with cytarabine was synergistic, causing reduced expression of the antiapoptotic genes *XIAP*, *BCL-2*, and *MCL-1*.

#### TG02

TG02 is an oral CDK9 inhibitor with activity against several CDKs in the nanomolar range [59]. TG02 exhibited potent antiproliferative effects against various tumor cell lines, induced cell-cycle arrest and apoptosis in murine mutant *FLT3* leukemia cells, and induced tumor regression and prolonged survival in murine AML models. In primary AML patient samples, TG02 inhibited transcription by inducing RNA Pol II Ser2 dephosphorylation and downregulated *MCL-1* and *XIAP*, leading to subsequent *BAX* activation and apoptosis [89]. Dynamic BH3 profiling has demonstrated that TG02 sensitizes to the BCL-2-inhibitory BAD-BH3 peptide in AML cells [68]. In addition, TG02 was shown to synergize with the BCL-2 antagonist venetoclax (ABT-199), which sensitizes to the MCL-1-inhibitory NOXA-BH3 peptide, to induce apoptosis in AML cells.

Phase 1 studies evaluating TG02 in advanced hematologic malignancies (ie, relapsed AML or ALL, chronic myeloid leukemia in blast crisis, or MDS; NCT01204164) and in R/R CLL or small lymphocytic lymphoma (NCT01699152), have been completed.

### CDK9 inhibitors in preclinical development in AML and other hematologic cancers

#### CDKI-73 (LS-007)

CDKI-73 is a potent CDK9 inhibitor (IC_50_ = 6 nM) that also displays strong activity against CDK1, CDK2, and CDK4 [51]. CDKI-73 inhibited phosphorylation of RNA Pol II Ser2 and transcription of *MCL-1* and *XIAP*, and induced apoptosis, in primary CLL cells and in AML and ALL cell lines [51, 52]. In CLL cells, CDKI-73 induced apoptosis via caspase-3 activation and displayed synergistic activity when combined with fludarabine, reversing the increase in MCL1 and XIAP seen with fludarabine alone [52]. CDKI-73 also decreased survival of primary AML and ALL cells and displayed synergism with the BCL-2 inhibitor ABT-199 against acute leukemia cell lines [51].

#### LY2857785

LY2857785 is a potent CDK9 inhibitor (IC_50_ = 11 nM) that also displays activity against CDK8, as well as CDK7 to a lesser degree [46]. LY2857785 was shown to inhibit Ser2 and Ser5 of RNA Pol II in primary AML and CLL cells and in an orthotopic leukemia model. It also inhibited cell proliferation of a variety of leukemia and solid tumor cell lines and reduced levels of MCL-1, resulting in apoptosis.

## Conclusions

Inhibition of CDK9 leads to selective downregulation of cell survival genes regulated by super enhancers and with short half-lives such as *MCL-1*, *MYC*, and cyclin D1. A variety of CDK9 inhibitors investigated in preclinical and clinical studies have demonstrated antiapoptotic and antitumor effects. However, the lack of selectivity for CDK9 may contribute to the less than optimal clinical efficacy and side effect profiles seen with CDK9 inhibitors thus far, necessitating investigation into more targeted approaches to improve outcome. Also the optimal pharmacokinetic profile and dosing schedule for CDK9 inhibitors is yet to be determined. In addition to use of predictive biomarkers, another rational approach is targeting multiple survival pathways, such as targeting both CDK9 and BRD4 to overcome increased *MYC* expression induced by CDK9 inhibition, or dual inhibition of both CDK9 and BCL family members [90, 91].

## Additional file

Additional file 1: **Table S1** CDK inhibitors utilized in clinical trials for the treatment of various types of malignancies. (DOCX 21 kb)

## Abbreviations

ALL: acute lymphoblastic leukemia
AM: cytarabine and mitoxantrone
AML: acute myeloid leukemia
ATL: adult T-cell leukemia/lymphoma
BCL-2: B-cell lymphoma 2
BH3: BCL-2 homology domain 3
CDK: cyclin-dependent kinase
CDK9_42_: 42 kDa isoform of CDK9
CDK9_55_: 55 kDa isoform of CDK9
CLL: chronic lymphocytic leukemia
CR: complete remission
CTD: carboxy-terminal domain
DLBCL: diffuse large B-cell lymphoma
FDA: US Food and Drug Administration
FLAM: flavopiridol, cytarabine, and mitoxantrone
HEXIM1: hexamethylene bisacetamide-inducible protein 1
HTLV-1: human T-lymphotropic virus-1
IC: inhibitory concentration
MDS: myelodysplastic syndrome
MM: multiple myeloma
mRNA: messenger RNA
P-TEFb: positive transcription elongation factor b
R/R: relapsed and/or refractory
RB: retinoblastoma
RNA Pol II: RNA polymerase II holoenzyme
Ser2/5: serine residues in peptide sequence YSPTSPS
TLS: tumor lysis syndrome
TST: timed sequential therapy
