# Supplementary material for: CDK9 inhibitors in acute myeloid leukemia
Source: J Exp Clin Cancer Res. 2018 Feb 23;37:36. doi: 10.1186/s13046-018-0704-8 (PMC5824552; doi:10.1186/s13046-018-0704-8)
Supplement: Supplementary file 1 — Table S1 CDK inhibitors utilized in clinical trials for the treatment of various types of malignancies. (DOCX 21 kb) [file 13046_2018_704_MOESM1_ESM.docx]

| Additional file 1: Table S1.  CDK inhibitors utilized in clinical trials for the treatment of various types of malignancies |
| --- |
| \|  \| \| \| \| \| --- \| --- \| --- \| --- \| \| CDK inhibitors (Alias[es]) \| Generation \| Principal antikinase activity \| Clinical trials for the treatment of tumors \| \|  \| \| \| \| \| Seliciclib (CYC-202;R-roscovitine) \| I \| CDK1, 2, 5, 7, 9; CK1; GSK3A; DYRK1A; ERK1 \| 3 studies. Phases I-II for NSCLC (terminated), breast cancer (withdrawn) and other solid tumors (recruiting) \| \|  \| \| \| \| \| Alvocidib (flavopiridol) \| I \| CDK1, 2, 4, 6, 7, 9; GSK3β \| 61 studies. Phases I-II for various types of hematologic and solid tumors. Many studies completed. Recruiting in AML \| \|  \| \| \| \| \| Dinaciclib (SCH727965) \| I \| CDK1, 2, 5, 9 \| 17 studies. Phases 1-2 for various solid tumors; phases 1-3 for hematologic cancers including completed phase 3 study in CLL. 3 studies recruiting. \| \|  \| \| \| \| \| SNS-032 (BMS-387032) \| I \| CDK1, 2, 4, 7, 9 \| 2 phase 1 studies, both completed, in B-cell malignancies and solid tumors. No active recruitment. \| \|  \| \| \| \| \| AG-024322 \| I \| CDK1, 2, 4, 7 \| 1 phase 1 study in non-Hodgkin’s lymphoma and solid tumors (terminated). No active recruitment. \| \|  \| \| \| \| \| R547 (R_0_-4584820) \| I \| CDK1, 2, 4, 7 \| 1 phase 1 study in solid tumors (completed). No active recruitment. \| \|  \| \| \| \| \| P276-00 \| II \| CDK1, 4, 9 \| 11 phase 1-2 studies, in hematologic and solid tumors. 7 studies completed. No active recruitment. \| \|  \| \| \| \| \| PD-0332991 (palbociclib) \| II \| CDK4, 6 \| 151 phase 1-4 studies, including across broad spectrum of hematologic and solid tumors . Regulatory approval achieved in breast cancer. Other CDK4/6 inhibitors, ribociclib and abemaciclib, have also achieved regulatory approval. \| \|  \| \| \| \| \| AT-7519 \| II \| CDK2, 4, 5, 9; GSK3β \| 5 phase 1-2 studies. 4 completed studies in different hematologic cancers. 1 phase 1 study recruiting in solid tumors. Phase 1-2a for advanced and/or metastatic solid tumors and refractory non-Hodgkin’s lymphoma \| \|  \| \| \| \| \| RGB-286638 \| II \| CDK1, 2, 4, 5, 7, 9 \| 1 phase 1 study in hematologic malignancies (withdrawn) \| \|  \| \| \| \| \| ZK 304709 \| III \| CDK1, 2, 4, 7, 9; VEGFR1, 2, 3;  PDGFR-b; Flt-3 \| No longer listed at clinical.trials.gov (closed in Phase I). \| \|  \| \| \| \| \| GPC-286199 (RGB-286199) \| III \| CDK1, 2, 3, 5, 7, 9; CRKs \| Preclinical stage. Not listed in clinical.trials.gov. \| \|  \| \| \| \| \| JNJ-7706621 \| III \| CDK1, 2, 3; Aurora A/B \| Preclinical stage. Not listed in clinical.trials.gov. \| |

CDK: cyclin-dependent kinase; CLL: chronic lymphocytic leukemia; CRK: CDK-related kinase; DYRK: dual-specificity tyrosine phosphorylation-regulated kinase; ERK: extracellular-signal related kinase; Flt: fms-like tyrosine kinase; NSCLC: non-small cell lung cancer; PDGFR-b: platelet-derived growth factor receptor beta; VEGFR: vascular endothelial growth factor receptor.

Adapted from Romano G. Deregulations in the cyclin-dependent kinase-9-related pathway in cancer: implications for drug discovery and development. ISRN Oncol. 2013;305371. Updates based on information in clinicaltrials.gov ([http://www.clinicaltrials.gov](http://www.clinicaltrials.gov/)).
